# Predictors of maternal role adaptation in Iranian women: a cross-sectional study

**DOI:** 10.1186/s12884-022-04702-2

**Published:** 2022-04-28

**Authors:** Parivash Ahmadpour, Shayesteh Jahanfar, Monireh Hamed Bieyabanie, Mojgan Mirghafourvand‬‬‬‬‬‬‬‬‬‬‬‬‬‬

**Affiliations:** 1 Midwifery Department, Tabriz University of Medical Sciences, Tabriz, Iran; 2 Department of Public Health and Community Medicine, MPH Program, Tufts University School of Medicine, Boston, USA; 3 Midwifery Department, MSc in Counseling in Midwifery, Tabriz University of Medical Sciences, Tabriz, Iran; 4 Social Determinants of Health Research Center, Nursing & Midwifery Faculty, Tabriz University of Medical Sciences, Tabriz, Iran

**Keywords:** Childbirth satisfaction, Self-confidence, Maternal adaptation

## Abstract

**Background:**

Numerous factors play a role in maternal identity formation and function. Recognizing the aspects related to adaptation to the mother role can effectively provide a solution to help mothers construct maternal roles. Consequently, this study aimed to determine the predictors of adaptation to the maternal role in Iranian women.

**Methods:**

This cross-sectional study was performed on 564 women who gave birth within one to four months after delivery with a record in Tabriz-Iran health centers, 2020–21. Participants were selected by cluster sampling. Data were collected using questionnaires of socio-demographic and obstetrics characteristics, Birth Satisfaction Scale-Revised (BSS-R), Lipz Maternal Self-Confidence Scale (LMSCS), and maternal role adaptation questionnaire. The general linear model was used to estimate the effect of each of the independent variables (socio-demographic and obstetrics characteristics, childbirth satisfaction, and self-confidence) on the dependent variable (maternal role adaptation).

**Results:**

The mean (± SD) total scores of adaptation to the maternal role, childbirth satisfaction, and maternal self-confidence were 77.4 (± 15.2) (score range: 33–165), 17.0 (± 5.9) (score range: 0–40), and 65.1 (± 13.5) (score range: 24–144), respectively. Based on the Pearson correlation test, there was a significant direct correlation between the overall score of adaptation to the maternal role with childbirth satisfaction (*r* = 0.462, *P* < 0.001) and maternal self-confidence (*r* = 0.652, *P* < 0.001). Based on the adjusted general linear model, the variables of maternal self-confidence, childbirth satisfaction, adequacy of household income, and spouse support were predictors of adaptation to the mother role. They explained 50.6% of the variance in the adaptation to the mother role score.

**Conclusions:**

Concerning the study results, adaptation to the maternal role is related to childbirth satisfaction and some socio-demographic variables. Therefore, considering the impact of maternal role on other aspects of women's life and child care, healthcare providers' and policymakers' critical role is to create positive childbirth experiences and strengthen mothers' self-confidence.

## Background 

Becoming a mother is associated with physical, social, and psychological changes that can affect the mother's mental health and all other aspects of her personal and family life [1, 2]. The role of the mother is one of the most basic and essential roles of women during their lives. The role of the mother means to create a motherly identity and perform actions based on the child's needs. With the acceptance of a new family member after childbirth, the role of the mother begins with the interaction between family members. It is strengthened by the growth of the child throughout the life cycle [3]. According to Thornton and Nardi's research, the development of maternal identity began during pregnancy, that is, psychosocial readiness to role-play begins, and at birth, continues by recognizing the baby that by copying the behaviors of others and following the recommendations. The mother's role is critical because it has a decisive effect on the child's life, develops the ideal relationship between mother and baby, and forms the child's natural development [4].

The mother's views on herself and her social and family roles change in the postpartum period [1, 2]. She has to take care of the baby while adapting to the new situation [5]. Henceforth, she faces many challenges through the process of adaptation [1]. Adaptation to maternal role is a reconstruction of the mother's sense of self. It includes establishing a responsible maternal role and conceptualizing that is known by the formation of maternal behaviors and the developement of a new identity [6]. According to Rubin (1984), adaptation to motherhood process begins in pregnancy and continues after delivery [7]. Most women achieve maternal identity up to four months after giving birth. Hence motherhood continues to evolve as a concept. According to Ruben and Mercer, transference to motherhood was smooth and without much difficulty for most women. Because mothers have a strong sense of responsibility to play the role of mother [8]. Usually, the transition to the maternal stage as a natural crisis causes significant adoption problems for most mothers and is a important issue for health providers [9].

Numerous variables have been identified, including maternal age, perception of the childbirth experience, preterm delivery, social stress, social support, and personality traits in the formation of maternal identity and functioning. Likewise, many social and psychological factors exist regarding maternal adjustment and factors that affected it [10, 11]. Other variables that have been identified as significant in achieving the role of a mother are as follows: the mother's own parenting experiences, her relationship with her spouse and previous experience with the children, and the events and policies of the mother's environment. Therefore, it is necessary to identify these variables that affect the mother's role in different cultures and societies. Mothers' self-confidence has been considered a key variable for adapting to the mother's role [12].

Mercer considers the mother's self-confidence as one of the emotional components of the mother's role, and at the same time thinks the mother's understanding of her ability to provide care for the child and to recognize and respond to the child's behavior while feeling satisfied with the mother's role [13]. Lederman et al. have indicated that self-confidence is an integral part of achieving motherhood [14]. If a woman feels that she can take good care of the child, her self-esteem will increase, and her interaction with her child will be positive, and she will show a loving reaction towards the child [4]. On the other hand, if the mother's confidence in caring for the child decreases, it becomes challenging to perform the role of the mother [3].

Childbirth satisfaction is an essential indicator of the quality of reproductive health services and maternal care. It has immediate and long-term effects on women's health and the quality of their relationship with the child [15]. Mothers with pleasant childbirth experiences have more self-esteem and have a stronger relationship with their children [16]. In contrast, dissatisfaction with childbirth increases the likelihood of postpartum depression, anxiety [17], post-traumatic stress disorder [18], impaired mother-infant bonding [15], fear of having the next child, and choosing to have a cesarean in the future [19]. Despite the extensive studies that have been done on women's childbirth experiences in different countries, there are still many unknown aspects in this stressful postpartum stage and the formation of maternal identity [20]. All these factors and features are influential in forming and adapting to the maternal role [8].

Iran is currently transitioning from medical to physiological delivery management. It tries to reduce unnecessary medical interventions, respect the mother's privacy, and use non-therapeutic methods to reduce pain, improve childbirth experiences and satisfaction [21]. Though, not many studies have been conducted on the effects of these factors on maternal role adaptation [22]. Recognizing the type of women's experiences of childbirth and the impact of these experiences on maternal roles can be a way to help mothers in the formation of maternal roles [3]. Awareness of the challenging experiences of childbirth on the construction and adaptation to the maternal role, while identifying the differences and complexities of such incidents, expands our knowledge of the reaction to these conditions [22]. Since the child's health and the quality of child care depend on the formation and adaptation to the role of the mother [15]; therefore, this study aimed to determine the predictors of adaptation to the maternal role.

## Methods

### Study design and participant

The current study is a cross-sectional study on 564 women referred to health centers in Tabriz-Iran in 2020–21. Inclusion criteria include women who gave birth within 1 to 4 months after delivery, having a record in Tabriz health centers, willingness to participate in the study, having a single child, and exclusion criteria: having any mental illness history according to the mother.

### Sampling

After the plan's approval by the research ethics committee of Tabriz University of Medical Sciences (Ethics code: IR.TBZMED.REC.1399.246), sampling began. In this study, 564 postpartum women were selected by cluster sampling from health centers in Tabriz. First, a quarter of Tabriz health centers were randomly selected using the website www.random.org. By referring to the centers chosen, the researcher extracted the list of women who gave birth 1 to 4 months after delivery. The number of selected samples from each center was determined based on the number of women covered by each center, then randomly selected from each center. Women were randomly selected, the selected individuals were contacted by phone, and while stating the objectives and method of the study briefly, they were invited to be at the health center on a particular day. In the face-to-face visit, the aims and process of the study were thoroughly explained, and written informed consent was obtained. Participants were reassured that their names and information would be kept confidential and that results would be reported anonymously. The data collection tool was then completed in a relatively quiet environment.

## Instruments

In this study, socio-demographic and obstetrics characteristics questionnaires, Birth Satisfaction Scale-Revised (BSS-R), Lipz Maternal Self-Confidence Scale (LMSCS), and Maternal Role Adaptation Questionnaire were used collect data.

Socio-demographic and obstetrics history included the following variables: age, age of spouse, age of marriage, marital status, housing status, monthly income adequacy for living expenses, level of education, level of education of spouse, job, occupation of spouse, body mass index, spouse support, family support, participation in pregnancy classes, timelag from the previous delivery, gestational age, parity, number of abortions, current pregnancy status of wanted or unwanted, type of delivery, infant hospitalization in intensive care unit and the presence of family members or a private midwife in the delivery room.

## BSS-R

The BSS-R tool was developed in 2014 by Martin et al. to measure maternal satisfaction with childbirth experience. This questionnaire consists of ten items and based on a 5-point Likert scale (in items 1, 3, 5, 6, 9, and 10, a score of 4 equals strongly agree and a score of zero equals strongly disagree, and in items 2, 4, 7 and 8 the scoring is reversed so that zero equals strongly agree and four equals strongly disagree). A score of zero is dissatisfaction, and 40 is the highest satisfaction. This scale has three subscales that include: experience of stress during labor (items 2, 1, 7, and 9), characteristics of women (items 4 and 8), and quality of care (items 3, 5, 6, and 10) [23]. This tool has been psychometric assessed in Iran by Nasiri et al. [24].

### LMSCS

This questionnaire has 24 items of self-report and assesses the mother's confidence in caring for the baby and recognizing the baby's needs. Each item is in 6 Likert scales from strongly agree to strongly disagree, with a score of 1 for strongly agree and 6 for disagree entirely. The questions of this questionnaire are written positively and negatively. A higher score indicates more self-confidence [25]. This tool has been validated in the study of Jafarnejad et al. in Iran [26].

### Maternal role adaptation questionnaire

Javadifar et al. developed this questionnaire in 2013. It includes 33 items, and its sub-domains include support and strengthening of couples' relationships (6 items), difficulty and dissatisfaction (7 items) and child attachment (4 items), worry and anxiety (4 items), performance and adjustment (4 items), emotional development (4 items) and social development (4 items). Each answer is based on a 5-point Likert scale, the scale is scored from strongly agree to disagree strongly, and the minimum and maximum scores were 33 and 165, respectively. A higher score indicates better adaptation. The content validity of this tool has been confirmed, and its Cronbach's alpha coefficient was 0.87 [27].

In the current study, the validity of the socio-demographic and obstetrics characteristics questionnaire was confirmed through content and face validity. The reliability of the questionnaires was confirmed by calculating Cronbach's alpha coefficient. For BSS-R equal to 0.83, Maternal self-confidence was equivalent to 0.79, and adaptation to the maternal role was equal to 0.87, respectively.

### Sample size estimation

The sample size was estimated based on the results of the study of Martin et al. [28] and taking into account the standard deviation (SD = 2.86), α = 0.05, and the accuracy of the study (d) equal to 0.05 around the mean (8.24) and power = 90% was equivalent to 376. Due to the cluster sampling and considering the design effect equal to 1.5, the final sample size was calculated to equal 564.

### Data analysis

Data analysis was done using SPSS version 25. Descriptive statistics, including frequency and percentage, mean and standard deviation, were used to describe the socio-demographic and obstetrics characteristics of the participants, the level of childbirth satisfaction, self-confidence, and adaptation to the mother role. The normality of quantitative data was measured using Skewness and Kurtosis, which had a normal distribution. Pearson correlation test was used to determine the relationship between childbirth satisfaction and self-confidence with adaptation to the maternal role. To determine the relationship between socio-demographic and obstetrics characteristics with the variable of transformation to the maternal role, a one-way analysis of variance and independent t-test were used. Then the general linear model was used to predict the effect of each of the independent variables (socio-demographic and obstetrics characteristics, childbirth satisfaction, and self-confidence) on the dependent variable (adaptation to the maternal role).

## Results

Five hundred sixty-four women who gave birth from November 2020 to March 20,201 were studied. The age of the participants was 29.5 (6.4) years. Less than a third of women (30.1%) had a university degree, and most (80.1%) were housewives. The mean age of the spouses was 33.8 (6.1), and about a third of them (32.8%) had a college education. Also, almost half of the spouses (45.4%) were shopkeepers or self-employed. More than half of the women (87.8%) reported that their monthly income was relatively sufficient. More than half of the participants (58.2%) lived in a private home. The majority (72.3%) stated that they received a lot of support from their spouse. About half of people (48.8%) experienced their first pregnancy (Table 1).

The mean (± SD) total scores of adaptation to the maternal role, childbirth satisfaction, and maternal self-confidence were 77.4 (± 15.2) (score range: 33–165), 17.0 (± 5.9) (score range: 0–40), and 65.1 (± 13.5) (score range: 24–144), respectively. Based on the Pearson correlation test, there was a significant direct correlation between the overall score of adaptation to the maternal role with childbirth satisfaction (*r* = 0.462, *P* < 0.001) and maternal self-confidence (*r* = 0.652, *P* < 0.001) (Table 2).

**Table 1:** Socio-demographic characteristics of the participants

Characteristics		Relation with adaptation to the maternal role	
	Number (Percent)	* Mean (SD)*	*P*-Value
**Age** (Year)	29.5 (6.4)^a^	-0.058^e^	0.172^d^
**Wife age** (Year)	33.8 (6.1)^a^	-0.023^e^	0.578^d^
**Marriage age** (Year)	23.0 (5.2) ^a^	-0.066^e^	0.118^d^
**Body mass index** (kg/m^2^)	27.5 (3.2) ^a^	0.068^e^	0.104^d^
**Sufficiency of income for expenses**			0.002^b^
Completely sufficient	104 (18.4)	75.0 (16.0)	
Somewhat sufficient	326 (57.8)	76.5 (14.5)	
Insufficient	134 (23.8)	81.4 (15.5)	
**Home status**			<0.001^b^
Private house	328 (58.2)	75.5 (14.9)	
Corporate home	203 (36.0)	80.8 (14.8)	
Living in a relatives' house	33 (5.9)	75.3 (16.4)	
**Education**			0.002^b^
Primary school	29 (5.2)	79.5 (18.2)	
Secondary school	96 (17.0)	78.5 (14.8)	
High school	111 (19.7)	81.2 (10.6)	
Diploma	158 (28.0)	77.4 (14.1)	
University	170 (30.1)	73.9 (17.5)	
**Spouse education**			0.007^b^
Illiterate	10 (1.8)	84.9 (13.3)	
Primary school	36 (6.4)	80.5 (17.3)	
Secondary school	66 (11.7)	76.8 (14.3)	
High school	74 (13.1)	80.1 (11.2)	
Diploma	193 (34.2)	78.6 (13.7)	
University	185 (32.8)	74.3 (17.4)	
**Job**			0.041^b^
Housewife	456 (80.9)	78.2 (14.6)	
Working at home	56 (17.0)	74.0 (17.8)	
Working abroad	12 (2.1)	74.5 (10.1)	
**Spouse employment**			0.001^b^
Unemployed	6 (1.1)	86.6 (12.2)	
Employee	119 (21.1)	73.4 (17.9)	
Manual worker	183 (32.4)	80.2 (14.6)	
Self-employment	256 (45.4)	77.0 (13.7)	
**Spouse support**			<0.001^b^
Low	24 (4.3)	94.6 (15.3)	
Moderate	132 (23.4)	80.9 (13.5)	
Much	185 (32.8)	78.8 (13.9)	
Very much	223 (39.5)	72.3 (14.9)	
**Family support**			<0.001^b^
Very Low	16 (2.8)	74.2 (23.4)	
Low	33 (5.9)	84.5 (17.0)	
Moderate	119 (21.1)	81.2 (13.4)	
Much	211 (37.4)	78.6 (13.7)	
Very much	185 (32.8)	72.6 (15.4)	
**Gestational age**	37.5 (2.8)		0.034^c^
≤ 37 (week)	124 (22.0)	77.2 (12.9)	
≥ 38 (week)	440 (78.0)	77.5 (15.7)	
**Gravid**	1.8 (1.0)		0.216^b^
1	275 (48.8)	77.0 (14.8)	
2	177 (31.4)	78.9 (15.3)	
≥ 3	112 (19.9)	75.9 (15.5)	
**Abortion**			0.573^c^
Yes	457 (81.0)	77.7 (15.4)	
No	76 (13.5)	76.2 (14.2)	
**Type of Pregnancy**			0.334^c^
Wanted	459 (81.4)	77.3 (15.5)	
Unwanted	1.5 (18.6)	78.0 (13.8)	
**Prenatal education**			0.458^c^
No	494 (87.6)	77.6 (14.9)	
Yes	70 (12.4)	76.1 (16.5)	
**Mode of Delivery**			0.274^b^
Normal vaginal delivery (NVD)	165 (29.2)	84.6 (10.4)	
Elective cesarean section	178 (31.6)	76.9 (17.0)	
Emergency cesarean section	221 (39.2)	76.7 (13.2)	
**Admission of newborn in NICU**			0.018^c^
No	77.6 (15.7)	77.6 (15.7)	
Yes	76.5 (12.5)	76.5 (12.5)	
**Presence of a doula during labor and delivery**			0.841^c^
No	77.5 (15.2)	77.5 (15.2)	
Yes	67.6 (12.2)	67.6 (12.2)	

^a^The numbers show mean (standard deviation)

^b^One-way ANOVA

^c^Independent t-test

^d^Pearson Correlation test

^e^Correlation coefficient

Based on the results of bivariate tests, there was a statistically significant relationship between the overall score of adaptation to the role of mother with the variables of housing status (*P*˂0.001), job (*P* = 0.041), spouse occupation (*P* = 0.001), household income adequacy (*P* = 0.002), spouse support (*P*˂0.001), family support (*P*˂0.001), education level (*P* = 0.002), spouse education level (*P* = 0.007), infant hospitalization in the neonatal intensive care unit (*P* = 0.018), and gestational age (*P* = 0.034). These variables were included in the general linear model and the variables of childbirth satisfaction and self-confidence as independent variables and adaptation to the maternal role as a dependent variable. The results of the adjusted general linear model showed that the variables of maternal self-confidence, childbirth satisfaction, adequacy of household income, and spouse support had a statistically significant relationship with the overall score of adaptation to the maternal role (*p*˂0.05), and were able to predict 50.6% of the variance of adaptation to the maternal role score. In women with higher childbirth satisfaction (B = 0.54; 95% CI: 0.36 to 0.71; *P* < 0.001) and higher maternal self-confidence (B = 0.58; 95% CI: 0.50 to 0.66; *P* < 0.001), the adaptation to the maternal role score was increased. Also, women with low spouse support had a higher adaptation to the maternal role score compared to women with high spouse support (B = 10.71; 95% CI: 5.73 to 15.71; *P* < 0.001), and in women with insufficient income compared to women with sufficient income (B = -3.74; 95% CI: -6.76 to -0.71; *P* = 0.022), the score of adaptation to motherhood was lower (Table 3).

**Table 2:** The statues of maternal role adaptation, childbirth satisfaction, self-confidence and their relationships in participants (*n* = 564)

Variable	Mean (SD^a^)	Obtainable score range	Obtained score range	Relationship with maternal self-confidence		Relationship with childbirth satisfaction	
				P^b^	r	P^b^	r
Maternal role adaptation	77.4 (15.2)	33 to 165	41 to 121	<0.001	0.652	<0.001	0.462
Childbirth satisfaction	17.0 (5.9)	0 to 40	3 to 38				
Maternal self-confidence	65.1 (13.5)	24 to 144	24 to 102				

^a^Standard deviation

^b^Pearson Correlation test

**Table 3:** Predictors of maternal role adaptation based on general linear model (*n* = 564)

Variable	B (CI 95%^a^)	*P*-value
**Childbirth satisfaction**	0.54 (0.31 to 0.72)	< 0.001
**Maternal self-confidence**	0.58 (0.50 to 0.66)	< 0.001
**Spouse support** (Reference: Very much)		
Much	0.99 (-1.53 to 3.52)	0.414
Moderate	2.78 (0.10 to 5.47)	0.032
Low	10.72 (5.73 to 15.71)	0.000
**Sufficiency of income for expenses** (Insufficient)		
Somewhat sufficient	-1.63 (-4.05 to 0.79)	0.194
Completely sufficient	-3.74 (-6.76 to -0.71)	0.022

^a^Confidence Interval

## Discussion

The present study results revealed a significant correlation between the overall score of adaptation to the maternal role, maternal self-confidence, and maternal satisfaction so that with increasing childbirth satisfaction and maternal self-confidence, adaptation to the maternal role also improved. Likewise, household income adequacy and spouse support variables were predictors of the adaptation to the maternal role.

One of the factors related to adaptation to the maternal role in the present study was childbirth satisfaction. A survey conducted by Kiehl et al. (2003) in Sweden, Norway, and the United States also indicated that more preparation for childbirth and satisfaction in the postpartum period increase adaptation with the maternal role [29], which is in line with the present study. Similarly in a study conducted by Javadifar et al. (2013) with the aim of internal conflicts of Iranian mothers in adapting to the maternal role qualitatively in Iran showed that the first days, weeks, and months after childbirth for new mothers in Iran is associated with stress and a lot of conflicts. Differences between mental expectations and postpartum experiences play an essential role in creating postpartum mental strife. These conflicts reduce the mother's ability to adapt to the maternal role [28]. The psychological health of mothers in the postpartum period is essential and can be examined to form and adjust to the maternal position. Consequently, despite improving the level of education and monitoring the physical dimension of maternal health during pregnancy and postpartum, the psychological measurement of women's health, prenatal experiences, women's mental image of motherhood, and related changes and problems should be given more attention [30].

In the current study, there was a significant relationship between self-confidence and adaptation to the maternal role. Mothers' self-confidence is vital for the transition to motherhood and the formation of the process of maternal identity. Emmanuel et al. state that successful adaptation to the mother's roles makes the mother feel more confident and more satisfied with her child care [2]. Also, a study conducted by Ha et al. (2013) in Korea to investigate the factors affecting maternal self-confidence in postpartum mothers revealed a significant negative relationship between child care stress and self-confidence and maternal role. As the stress score increases, the score of self-confidence and maternal role decreases [31]. Low maternal self-confidence leads to delays in forming maternal roles and identity, causing incompatibility with the maternal role. This non-adaptation to the maternal role leads to a decrease in mothers' satisfaction with their maternal role. However, mothers with high self-esteem find it easier to achieve maternal roles and satisfaction with their roles [26], consistent with the present study's findings.

In the current study, adapting to the maternal role was better for those who received less support from their spouse than those who received more support. A survey conducted in Sari, Iran, to examine the factors affecting adaptation to the maternal role of nulliparous women in the first month to one year after childbirth revealed that spouse support had a statistically significant effect on adaptation to the maternal role. So that women who received more support from their husbands had better adaptation to the maternal role [32]. Likewise, a descriptive prospective study in Australia aimed at shaping the maternal role in pregnant women [36 weeks pregnancy and 12 weeks postpartum] showed that social support has a statistically significant effect on adaptation to women's maternal role [2]. Mothers need emotional support after childbirth. These supports can help mothers' health as well as their role as mothers in the postpartum period. This support can be provided by a spouse, other relatives, or health workers [13]. The discrepancy in the study findings can be attributed to changes in the needs of children at different ages. For example, within one year of giving birth, most women accept their maternal roles and responsibilities and acquire sufficient information about their environment, which in turn increases their adaptation to the maternal role [33]. Differences in the findings of these studies and our research may also be due to differences in the tools for assessing the support. Many factors are recognized to impact on women's transition to motherhood. Social support appears to be highly valued by mothers. However, little is known regarding the mediating effect of social support on adaptation to motherhood [8]. In Cupples et al.’s study, despite possible longer-term social advantage, social support showed no benefit for infant development or maternal health at 1 year [34].

In the present study, mothers in families with an insufficient monthly income had better adaptation to the maternal role. The results of Ha et al.’s study showed that the higher the economic level is, the higher the adaptation to the maternal role is [31]. A survey by Hamzehgardeshi et al. (2018) [32] was done on the factors influencing adaptation to the maternal part of mothers in the postpartum period up to one year after birth. Economic status was determined as an essential predictive component and revealed that mothers with a higher financial situation are better adapted to the maternal role. While in other studies [35, 36], no significant relationship was found between economic status and adaptation to motherhood. Adaptation to motherhood is an emotional dimension, and due to the importance of motherhood, factors such as economics do not seem to impact women's maternal identity significantly. Though, more research is needed to clarify this issue [13]. According to Mangham et al. (2014), low- and middle-income countries have added routine public health services including breastfeeding practices, neonatal care and infant care in their routine programs to promote neonatal care and maternal function [37]. In Iran, as a middle-income country, maternal and newborn health services are provided for mothers in health centers for free. Thus, mothers in in families with an insufficient monthly income can receive all services and this may lead to better maternal role adaptation in this social class.

Due to its cross-sectional nature, one of the limitations of this study is that the relationships shown between adaptation to maternal role and satisfaction with childbirth and self-confidence and some socio-demographic characteristics do not accurately indicate a causal relationship. Similarly, the current study was done on COVID-19 pandemic conditions. The negative burden of these pandemic conditions affects the psyche of humans, especially mothers in the postpartum period [30]. The use of standard tools is one of the strengths of this study. Likewise, another strength of this research is random sampling, which causes a high degree of generalizability of the study. Still, since culture affects the adaptation to the maternal role [31], regarding the multicultural context of Iran, it is suggested that further research be conducted to explore the concept of maternal role adaptation in other provinces of Iran.

## Conclusion

Based on the results of the study, adaptation to the maternal role is related to childbirth satisfaction, maternal self-confidence, and some socio-demographic variables; consequently, considering the impact of maternal role on other aspects of female life and child care, by better caring for mothers during childbirth and creating positive childbirth experiences, women can better prepare for the challenges of the postpartum period and the role of motherhood. This fact identifies the central role of midwives, midwifery educators, and caregivers in helping to create positive childbirth experiences tailored to mothers' mental and psychological needs.

## Data Availability

Datasets used and analyzed during this study are available from the corresponding author on reasonable request.

## Abbreviations

BSS-RI: The Birth Satisfaction Scale-Revised Indicator
LMSCS: Lipz Maternal Self-Confidence Scale
COVID-19: Coronavirus Disease 2019
